# Biodiversity and Conservation Challenges in the Alédjo Wildlife Reserve (AWR) in Togo: Insights From Ethnozoological Surveys

**DOI:** 10.1002/ece3.72922

**Published:** 2026-01-07

**Authors:** Wiyaou Borozi, Wouyo Atakpama, Delagnon Assou, Armand Kuyema Natta

**Affiliations:** ^1^ Laboratory of Ecology, Botany and Plant Biology (LEB), Faculty of Agronomy University of Parakou Parakou Benin; ^2^ Laboratory of Botany and Plant Ecology (LBEV), Department of Botany, Faculty of Sciences University of Lomé Lomé Togo; ^3^ West Africa Plant Red List Authority (WAPRLA) IUCN Species Survival Commission Gland Switzerland; ^4^ Laboratory of Ecology and Ecotoxicology (LaEE), Department of Zoology and Animal Biology, Faculty of Sciences University of Lomé Lomé Togo

**Keywords:** Alédjo Wildlife Reserve, protected area, species use, species vulnerability, Togo, wildlife resources

## Abstract

The decline of forest ecosystems due to anthropogenic pressures directly threatens the wildlife that depends on them. These pressures extend even to protected areas, undermining their role as sanctuaries for animal species. Analyzing local knowledge and the vulnerability of wildlife species in the Alédjo Wildlife Reserve (AWR) in Togo can lead to improved research approaches and sustainable management strategies for this protected area. Semistructured ethnozoological surveys, including individual interviews and focus group discussions, were conducted with 298 people living near the AWR. Data analysis was based on wildlife species use indices and a vulnerability index to assess usage impacts. A total of 49 species, grouped into 46 genera and 31 families, were recorded. The most diverse families were Bovidae (5 species), Cercopithecidae (4 species), and Muridae (4 species). The Importance Value‐in‐use Index (IVIUsp) indicates that the most valued species are the patas monkey ( *Erythrocebus patas* ), the forest cobra ( *Naja melanoleuca* ), and the green mamba ( *Dendroaspis viridis* ). According to the International Union for Conservation of Nature (IUCN) Red List categories, nine species are globally threatened. Fourteen species are moderately vulnerable to local exploitation. Protecting the AWR ecosystems, which provide habitat and food resources for wildlife species, is crucial for their conservation. In addition, the study highlights the need for integrating both community livelihoods and conservation priorities to safeguard wildlife populations in AWR.

## Graphical abstract

The present study analyzes wildlife species use indices and a vulnerability in the Aledjo Wildlife Reserve (AWR) in Togo. The Importance Value‐in‐use Index (IVIUsp) indicates that the most valued species are the patas monkey ( *Erythrocebus patas* ), the forest cobra ( *Naja melanoleuca* ), and the green mamba ( *Dendroaspis viridis* ). Fourteen wildlife species were highlighted as moderately vulnerable to local exploitation.

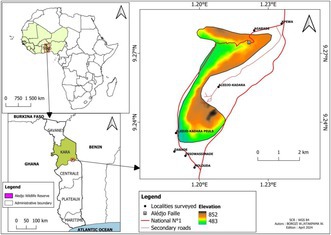

## 1 Introduction

Like most countries of Afrotropical forest ecosystems (Escobar et al. 2015; Tarimo et al. 2015; Adama et al. 2023), Togo is facing significant degradation and deforestation of its forest resources (Folega 2019; Hlovor et al. 2021; Kombate et al. 2022). This degradation has affected even the country's protected areas, which were once relatively well‐preserved (Folega et al. 2012; Polo‐Akpisso et al. 2018; Kokou et al. 2023). The degradation of these protected areas dates back to the socio‐political upheavals of the 1990s, impacting nearly all 83 protected areas (PAs) established in the country since the colonial era (MERF 2020). The vulnerability of forest ecosystems in these protected areas, which serve as preferred habitats for wildlife species (Issifou et al. 2023), impacts both animal population sizes and species diversity (Agbessi et al. 2017; Segniagbeto et al. 2022).

Several factors contribute to the degradation of wildlife habitats and the vulnerability of species in protected areas, including human pressures from population growth and political changes since the 1990s (Kokou et al. 2023). Numerous studies have reported disturbances and alarming degradation of wildlife habitats in protected areas due to anthropogenic pressures (Dimobe et al. 2012; Adjonou et al. 2017; Polo‐Akpisso et al. 2018; Badjare et al. 2021; Atakpama et al. 2023; Kombate et al. 2024). Other studies have shown that wildlife resources are often overexploited and sold in markets bordering protected areas (D'Cruze, Assou, Coulthard, Norrey, et al. 2020; Sonhaye‐Ouyé et al. 2022). While wildlife exploitation provides essential protein and income for local communities, it also leads to numerous violations within protected areas. As a result, many threatened and endemic species that once thrived in these refuges are now at risk of local extirpation (D'Cruze, Assou, Coulthard, Norrey, et al. 2020; D'Cruze, Harrington, Assou, Ronfot, et al. 2020; Assou et al. 2021; Segniagbeto et al. 2022).

The management approach before the 1990s, which excluded local communities involvement, is often cited as the main cause of the invasion and degradation of protected areas in Togo (Kokou et al. 2023). However, there is a limited number of studies on the vulnerability of Togo's wildlife to anthropogenic pressures (Issifou et al. 2022). Assessing the vulnerability of wildlife species in Togo's protected areas remains an understudied area, yet such knowledge is crucial for implementing effective management measures. These measures include increased surveillance, strict enforcement of wildlife protection laws, and raising awareness among local communities of new management paradigms.

Despite anthropogenic pressures, some protected areas in Togo's Central region, including the Alédjo Wildlife Reserve (AWR), remain viable with diverse wildlife and plant species (Woegan et al. 2013; MERF 2020). The diversity of ecosystems, flora, and fauna, along with attractive mountainous terrain, makes this protected area a valuable ecotourism attraction site (MERF 2017). Beyond tourism, preserving the biodiversity of this protected area, particularly its wildlife, is essential. The area is surrounded by several communities that heavily depend on its forest products (Wala et al. 2012). Exploitation of both timber and non‐timber forest products provides significant income (Dourma et al. 2009; Kaina et al. 2021) and food for local communities. However, this exploitation also contributes to the degradation of forest ecosystems and poses a threat to plant and animal diversity (Fontodji et al. 2011; Kaina et al. 2018).

The present study is based on the hypothesis that human pressures threaten the survival of AWR wildlife species, with vulnerability increasing with usage pressure. It aimed to address two main research questions: What are the diversity and use‐importance value of the AWR's wildlife resources? What is the level of wildlife vulnerability in the AWR in the face of anthropogenic pressures?

The general objective of this study is to evaluate local knowledge of wildlife uses and the vulnerability of species in the AWR in Togo. Specifically, it aimed to: (i) determine the diversity and use‐importance value of wildlife species in the AWR and (ii) assess the level of wildlife vulnerability to usage pressures.

## 2 Methods

### 2.1 Study Area

**FIGURE 1:**
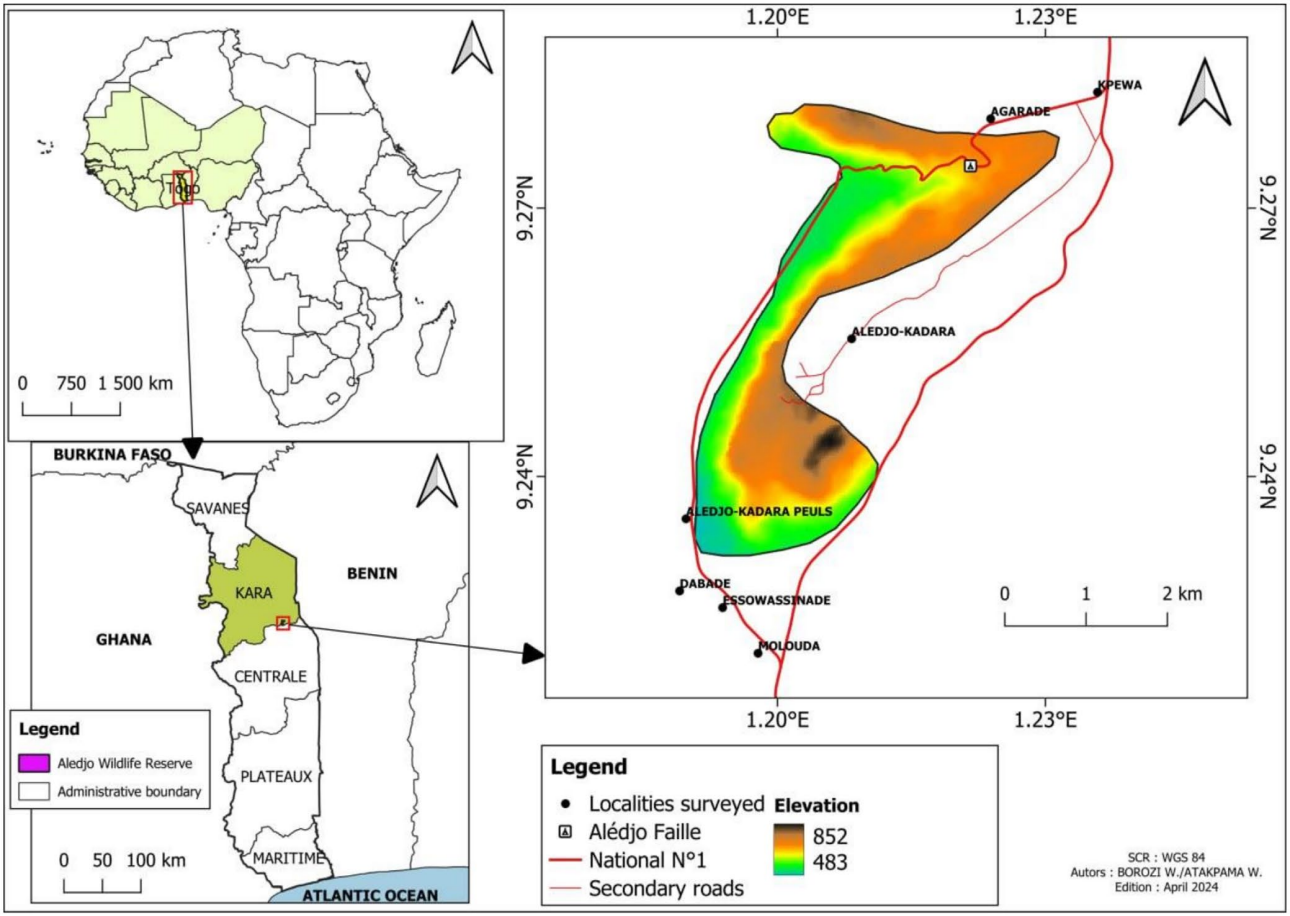
Location of the Alédjo Wildlife Reserve (AWR) in Togo, West Africa.

With an area of 765 ha, the Alédjo Wildlife Reserve (Figure 1) is located between the prefectures of Tchaoudjo in the Central region and Assoli in the Kara region (MERF 2017). The reserve is located between latitudes 1°23′ N and 1°29′ N and longitudes 1°18′ E and 1°24′ E. It was officially designated as a protected area by decree No. 411/EF on July 30, 1939. Located in ecological zone II of Togo, it experiences a tropical Sudano‐Guinean climate (Ern 1979) with an average annual temperature of 27.2°C and an average annual precipitation of 1298 mm. The mountainous terrain provides a panoramic view of diverse vegetation and geological formations. A notable feature within the reserve is the “Faille d'Alédjo”, a rock formation which was cut into two to make way for the N1 national road (Lomé‐Cinkassé) during the German colonization. The reserve hosts various tree species that are exploited by the local communities for firewood, timber, and service wood (Dourma et al. 2009; Wala et al. 2012; Kaina et al. 2021). Its wildlife includes duikers, bushbucks, primates, and a diverse array of bird species (MERF 2017).

### 2.2 Data Collection

Semi‐structured ethnozoological surveys, comprising individual interviews and focus group discussions (Issifou et al. 2022), were conducted among local populations living around the AWR. These surveys involved 298 participants and were carried out over two periods: August 2–13, 2022, and April 16–18, 2023. The participants were from six localities bordering the AWR: Alédjo Kadara, Alédjo Bas, Aléhéridè, Essowazina, Kpéwa, and Agaradè. Participation was open to individuals of all sexes, ages, and ethnicities (Badjare et al. 2021), including local chiefs, members of the protected area management committee, farmers, hunters, and housewives. Participants were recruited through targeted snowball sampling, starting with an influential community member (e.g., the Chief or the president of the hunters' group) who referred suitable interviewees able to provide information on the protected area and its wildlife. Precautions were taken to ensure the anonymity of all the respondents (e.g., names, location, and contact were not recorded). Participation is entirely voluntary, and the respondents were free to withdraw at any time without giving any reason. The interviews were relatively simple, lasting an average of 10–15 min per participant.

The surveys sought information on common animal species, the parts used, and the types of use, categorized as food, medicinal, cosmetic, traditional and cultural, artisanal, and commercial. The relative availability of the reported species was assessed using the pebble technique, with respondents categorizing species as very abundant (more than 75% available), abundant (50%–75% available), not abundant (25%–50% available), rare (less than 25% available), and very rare (barely existing). Reported species were confirmed through images of animals, hunting trophies, samples of hunted animals or live specimens provided by respondents. In addition to these data, sociodemographic information was collected, including gender, age, education level, and occupation of the respondents. The questionnaires were designed and deployed using the Kobotoolbox platform, with the Kobocollect mobile application used for field data collection.

Species identification was based on reference guides for the systematics and identification of West African and Togolese wildlife (Granjon and Duplantier 2009; Segniagbeto et al. 2011, 2017, 2018; Amori et al. 2016; Trape 2023). Each species was classified according to its family, order, and class, with reference to the aforementioned documents and the IUCN Red List (https://www.iucnredlist.org/).

A total of 82.55% (*n* = 246) were men and 17.45% (*n* = 52) were women. Fewer women participated in the survey due to gender roles: men are more involved in wildlife‐related activities, while women focus on farming and domestic tasks. The dominant ethnic group was the Tem (79.87%, *n* = 238), who are indigenous to the area. Other, less represented groups included the Kabyè (10.07%, *n* = 30), Fulani (7.05%, *n* = 21), and Moba (3.02%, *n* = 9). Islam was the most widely practiced religion (63.09%, *n* = 188), followed by Christianity (26.86%, *n* = 80), and animism (10.07%, *n* = 30). The average age of participants was 31 years.

### 2.3 Data Processing

#### 2.3.1 Assessment of the Diversity and the Use‐Importance Value of the AWR'S Wildlife

The data collected were processed using the Microsoft Excel spreadsheet. The assessment of the use value of AWR wildlife species was based on the Importance Value‐in‐use Index (IVIUsp). The IVIUsp is the sum of the Citation Frequency (CFsp), the Use Value of the species (UVsp), and the Use Diversity Index of the species (UDIsp): IVIUsp = CFsp + UVsp + UDIsp (Avocèvou‐Ayisso et al. 2011; Gadikou et al. 2022). This index makes it possible to better classify wildlife species of AWR based on its knowledge among respondents, the use of its different parts or derivatives, and the diversity of its uses.

The Citation Frequency (CFsp = P1, %) of species i: P1 = (𝑛𝑖/N) × 100 with *ni*, the number of respondents who cited it and N, the total number of respondents (Issifou et al. 2022). The vulnerability of a species decreases when its frequency decreases.

The Use Value of species (UVsp) is the ratio between the number of citations of the species or the Reported Use (RUsp) and the total sum of the numbers of uses of all species: ${\mathrm{UV}}_{\mathrm{sp}}=\frac{{\mathrm{RU}}_{\mathrm{sp}}}{\sum_{1}^{n}{\mathrm{RU}}_{\mathrm{sp}}}\times 100$. The species with the highest UVsp is the one whose use is most recognized. The reported use is the sum of the number of usage citations per part (NCp) of the species by the respondents: RUsp = ∑NCpp.

The Use Diversity Index (UDIsp) is the ratio between the number of specific uses of species i and that of the species having the maximum number of specific uses: $\text{UDIsp}=\frac{{\mathrm{NSU}}_{i}}{\sum {\mathrm{NSU}}_{\max}}\times 100$. The UDIsp of the most used species is 100.

#### 2.3.2 Vulnerability Assessment of the Usual Wildlife of the AWR

The assessment of the species vulnerability index was inspired by previous studies on flora (Traore et al. 2011; Amegbenyuie et al. 2023). The parameters used were adapted to the animal species. The vulnerability scale has three levels, from 1 to 3 (Betti 2001) (Table 1). A value of 1 designates a species with low vulnerability for the parameters indicated, 2 represents medium vulnerability, and 3 characterizes a highly vulnerable species. The assessment of species vulnerability was based on three (3) parameters: the Citation Frequency (CFsp = P1), the typology of specific uses (P2), and the presence indices reported (P3). The wildlife species Citation Frequency is the ratio between the number of people having cited the species and the total number of people interviewed during the investigation. The vulnerability of a species increases as the number of users increases (P1). The greater the number of uses, the more pressure is exerted on the species (P2). The vulnerability of a species increases when its availability decreases (P3). This availability is determined from the calculation of the consensus value (Issifou et al. 2022). The average of these three parameters corresponds to the species vulnerability index. Its value varies between 1 and 3. The three used parameters are presented in Table 1.

**TABLE 1:** Parameters used for calculating the vulnerability index.

Retained parameters	Low (scale = 1)	Average (scale = 2)	Strong (scale = 3)
Citation frequency: P1	P1 < 5%	5% ≤ P2 < 15%	P3 ≥ 15%
Use categories: P2	*p* < 2	2 ≤ P2 ≤ 3	P2 ≥ 4
Presence index: P3	Very abundant, Abundant	Scarce	Rare, very rare

The calculation of the species vulnerability index (VI) follows the following formula: $\mathrm{VI}=\frac{P1+P2+P3}{3}.$ If VI < 2, the species is considered to be less vulnerable. The species is considered to be moderately vulnerable if 2 ≤ VI < 2.5. It is considered to be vulnerable if VI ≥ 2.5. In addition to the assessment at the scale of the study area, the global conservation status of the species according to the IUCN Red List threat categories was recorded (IUCN 2022).

## 3 Results

### 3.1 The Use‐Importance Value of Wildlife Species in the AWR

**FIGURE 2:**
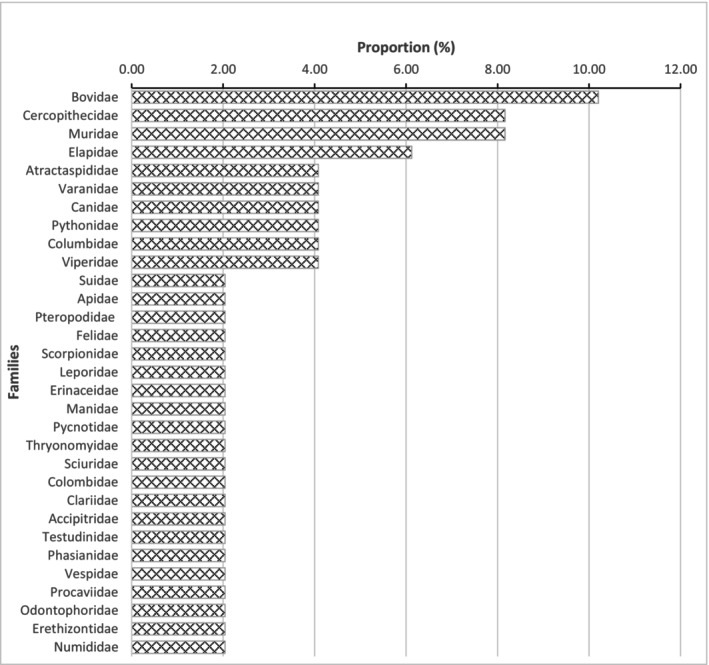
Proportion of the usual wildlife families of the Alédjo Wildlife Reserve in Togo.

During the investigations, 49 wildlife species were recorded in the AWR. These species are classified into 46 genera and 31 families. The most diverse family is Bovidae with 5 species, followed by Cercopithecidae and Muridae with 4 species each, and Elapidae with 3 species. The families Atractaspididae, Canidae, Columbidae, Sciuridae, Varanidae, and Viperidae each include two species. All other families are represented by a single species (Figure 2).

**FIGURE 3:**
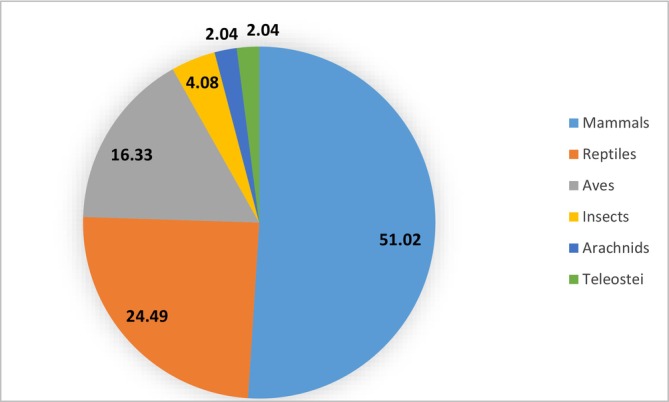
Distribution of recorded wildlife species classes.

The 49 recorded species belong to six classes, with Mammals, Reptiles, and Aves (birds) being the most significant. Mammals account for slightly less than half of the reported species (Figure 3). There are six ungulates represented by the Bovidae and four primates. Table 2 presents the distribution of the reported species by class, order, and family.

**TABLE 2:** Distribution of class, order, and family of the recorded wildlife species in the AWR.

Class	Order	Family	Scientific name	Common name
Teleostei	Siluriformes	Clariidae	*Clarias* spp.	Catfish
Arachnids	Scorpions	Scorpionidae	*Pandinus imperator*	Emperor Scorpion
Aves	Accipitriformes	Accipitridae	*Accipiter melanoleucus*	Black Sparrowhawk
	Galliformes	Phasianidae	*Pternistis bicalcaratus*	Double‐spurred Francolin
		Numididae	*Numida meleagris*	Helmeted Guineafowl
		Odontophoridae	*Ptilopachus petrosus*	Stone Partridge
	Passeriformes	Pycnonotidae	*Pycnonotus barbatus*	Common Bulbul
	Columbiformes	Columbidae	*Streptopelia semitorquata*	Red‐eyed Dove
		Columbidae	*Streptopelia vinacea*	Vinaceous Dove
		Columbidae	*Treron calvus*	African Green‐pigeon
Insecta	Hymenoptera	Apidae	*Apis mellifera*	Honey Bee
		Vespidae	*Eumenes pedunculatus*	Mason wasp
Mammals	Artiodactyls	Bovidae	*Philantomba walteri*	Walter's Duiker
		Bovidae	*Cephalophus rufilatus*	Rufous‐sided cephalopod
		Bovidae	*Kobus kob* ssp. *kob*	Buffon's Cob
		Suidae	*Phacochoerus africanus*	Warthog
		Bovidae	*Tragelaphus scriptus*	Harnessed bushbuck
		Bovidae	*Sylvicapra grimmia*	Grimm's Duiker
	Carnivores	Canidae	*Canis aureus*	Jackal
		Felidae	*Felis silvestris*	Wild cat
		Canidae	*Lycaon pictus*	Lycaon
	Bats	Pteropodidae	*Eidolon helvum*	Bat
	Eulipotyphla	Erinaceidae	*Atelerix albiventris*	Hedgehog
	Hyracoides	Procaviidae	*Procavia capensis*	Rock Hyrax
	Lagomorphs	Leporidae	*Lepus microtis*	Hare
	Pholidotes	Manidae	*Phataginus tricuspis*	Pangolin
	Primates	Cercopithecidae	*Cercopithecus petaurista* spp petaurista	White‐nosed Cercopithecus
		Cercopithecidae	*Chlorocebus tantalus*	Vervet monkey
		Cercopithecidae	*Erythrocebus patas*	Patas monkey
		Cercopithecidae	*Papio anubis*	Olive Baboon
	Rodents	Muridae	*Lemniscomys zebra*	Zebra rat
		Erethizontidae	*Hystrix cristata*	Porcupine
		Muridae	*Mus mattheyi*	Matthey's dwarf mouse
		Muridae	*Mus minutoides*	Pan‐African dwarf mouse
		Sciuridae	*Xerus erythropus*	Ground squirrel
		Muridae	*Rattus rattus*	Rat
		Thryonomyidae	*Thryonomys swinderianus*	Grasscutter
Reptiles	Squamates	Atractaspididae	*Atractaspis aterrima*	Slender atractaspide
		Atractaspididae	*Atractaspis dahomeyensis*	Atractaspide from Dahomey
		Viperidae	*Bitis arietans*	Puff adder
		Elapidae	*Dendroaspis viridis*	Green Mamba
		Viperidae	*Echis ocellatus*	Echis
		Elapidae	*Naja melanoleuca*	Forest cobra
		Elapidae	*Naja nigricollis*	Black‐necked spitting cobra
		Pythonidae	*Python regius*	Ball python
		Pythonidae	*Python sebae*	Seba Python
		Varanidae	*Varanus niloticus*	Water Monitor
		Varanidae	*Varanus exanthematicus*	Earth monitor lizard
	Testudines	Testudinidae	*Kinixys homeana*	Home Kinixys

The Importance Value‐in‐use Index (IVIUsp) shows five species of great importance for the local communities living around the AWR (Table 3). These are: the patas monkey ( *Erythrocebus patas* ), forest cobra ( *Naja melanoleuca* ), green mamba ( *Dendroaspis viridis* ), ball python ( *Python regius* ), and puff adder ( *Bitis arietans* ) with IVIUsp values ranging between 95.60 and 115.35. The importance of these species is justified by the number of respondents who reported the species, their number of citations of the species, and the diversity of specific uses of the species.

**TABLE 3:** The use‐importance value of the most important animal species of AWR.

Scientific name	FCsp	UVsp	DUIsp	IUVIsp
*Erythrocebus patas*	47.06	1.62	66.67	115.35
*Naja melanoleuca*	5.88	4.32	100.00	110.21
*Dendroaspis viridis*	35.29	3.24	66.67	105.20
*Python regius*	23.53	9.19	66.67	99.39
*Bitis arietans*	23.53	5.41	66.67	95.60
*Lycaon pictus*	47.06	2.70	33.33	83.09
*Tragelaphus scriptus*	47.06	2.70	33.33	83.09
*Xerus erythropus*	35.29	3.24	33.33	71.87
*Sylvicapra grimmia*	29.41	6.49	33.33	69.23
*Cephalophus rufilatus*	29.41	6.49	33.33	69.23
*Cercopithecus petaurista petaurista*	29.41	3.78	33.33	66.53
*Hystrix cristata*	29.41	1.08	33.33	63.83
*Marmota mumbled*	29.41	0.54	33.33	63.29
*Numida meleagris*	29.41	0.54	33.33	63.29
*Python sebae*	29.41	4.32	33.33	67.07
*Clarias* spp.	23.53	1.08	33.33	57.94
*Rattus rattus*	17.65	6.49	33.33	57.47
*Lemniscomys zebra*	23.53	0.54	33.33	57.40
*Mus mattheyi*	23.53	0.54	33.33	57.40
*Treron calvus*	17.65	1.62	33.33	52.60
*Ptilopachus petrosus*	17.65	1.08	33.33	52.06
*Eumenes pedunculatus*.	17.65	0.54	33.33	51.52
*Pycnonotus barbatus*	17.65	0.54	33.33	51.52
*Atelerix albiventrix*	11.76	2.70	33.33	47.80
*Philantomba walteri*	11.76	2.70	33.33	47.80
*Phacochoerus africanus*	11.76	0.54	33.33	45.64
*Lepus microtis*	5.88	5.95	33.33	45.16
*Naja nigricollis*	5.88	4.32	33.33	43.54
*Thryonomys swinderianus*	5.88	3.78	33.33	43.00
*Francolinus bicalcaratus*	5.88	2.70	33.33	41.92
*Kobus kob* ssp. *kob*	5.88	2.70	33.33	41.92
*Kinixys homeana*	5.88	1.08	33.33	40.30
*Papio anubus*	5.88	1.08	33.33	40.30
*Varanus exanthematicus*	5.88	1.08	33.33	40.30
*Varanus niloticus*	5.88	0.54	33.33	39.76
*Accipiter melanoleucus*	5.88	0.54	33.33	39.76
*Apis mellifra*	5.88	0.54	33.33	39.76
*Atractaspis aterrima*	5.88	0.54	33.33	39.76
*Atractaspis dahomeyensis*	5.88	0.54	33.33	39.76
*Canis aureus*	5.88	0.54	33.33	39.76
*Chlorocebus tantalus*	5.88	0.54	33.33	39.76
*Eidolon helvum*	5.88	0.54	33.33	39.76
*Felis silvestris*	5.88	0.54	33.33	39.76
*Mus minutoides*	5.88	0.54	33.33	39.76
*Pandinus imperator*	5.88	0.54	33.33	39.76
*Phataginus tricuspis*	5.88	0.54	33.33	39.76
*Streptopelia semitorquata*	5.88	0.54	33.33	39.76
*Streptopelia vinacea*	5.88	0.54	33.33	39.76

Abbreviations: DUIsp, diversity use value index; FCsp, frequency of citations; IUVIsp, importance use value index; UVsp, use value.

**FIGURE 4:**
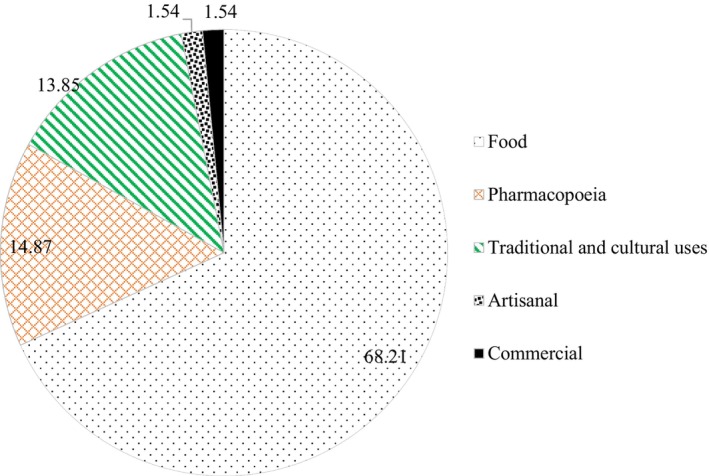
Typology of uses of wildlife species by local communities in the AWR.

Local communities utilize wildlife species primarily in five ways. The most predominant use is for food, accounting for 67.86% of all uses, indicating that wild meat consumption is popular. Secondary uses include pharmacopeia and traditional/cultural purposes. Commercial and artisanal uses are less frequently reported. Selling live animals as wild meat or pet trade is uncommon (Figure 4).

### 3.2 Vulnerability of AWR Wildlife Species

#### 3.2.1 Vulnerability of Wildlife Species of AWR According to IUCN Criteria

With regards to their international conservation status, nearly three quarters of the species reported in the AWR (74.51%) are classified as Least Concern on the IUCN Red List. Five species (09.80%) are Near‐Threatened ( *Cercopithecus petaurista* spp. *petaurista*, *Eidolon helvum* , *Erythrocebus patas* , *Python regius* , and *Python sebae* ), two are Critically Endangered ( *Kinixys homeana* and *Lycaon pictus* ), one is Endangered (*Phataginus tricuspis*) and one is Vulnerable ( *Kobus kob* ssp. *kob*). Additionally, there are two Data‐Deficient species ( *Apis mellifera* and *Philantomba walteri*). Two species (*Eumenes pedunculatus* and *Pandinus imperator*) have not yet been assessed against the IUCN Red List categories and criteria.

#### 3.2.2 Vulnerability of Wildlife Species to Local Exploitation in the AWR

**FIGURE 5:**
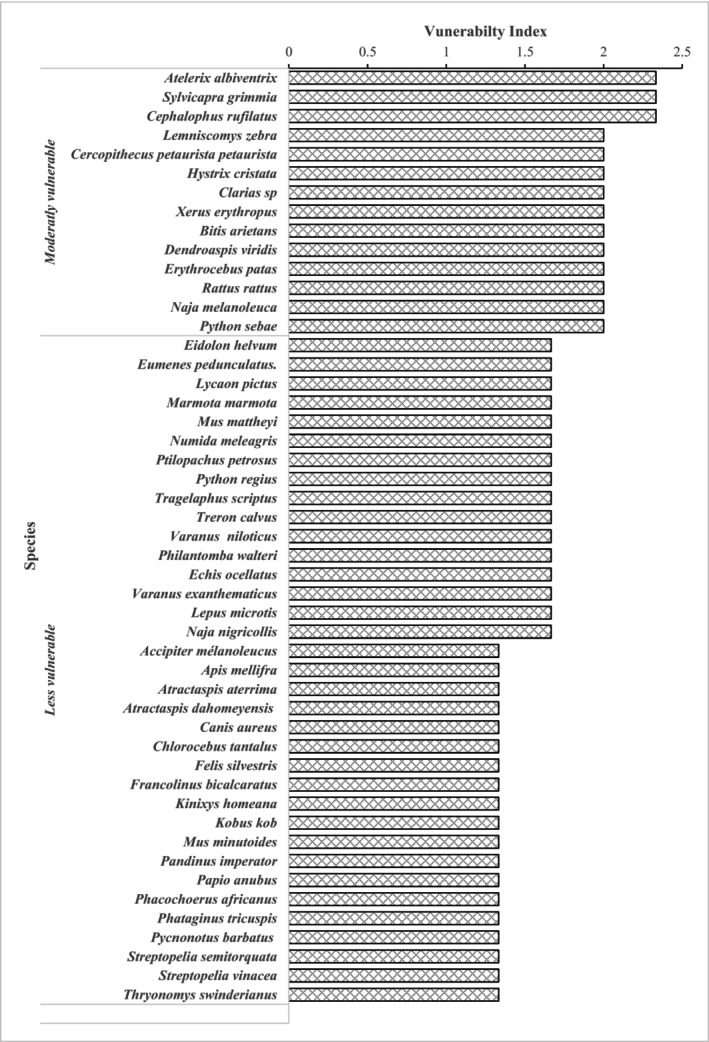
Vulnerability of wildlife species to local exploitation in the AWR.

The results show that no species are categorized as highly vulnerable to local exploitation. However, 14 species are classified as moderately vulnerable (Figure 5). These include *Atelerix albiventrix*, *Sylvicapra grimmia* , *Cephalophus rufilatus, Lemniscomys zebra*, *Cercopithecus petaurista* spp. *petaurista*, *Hystrix cristata* , *Clarias* spp., *Xerus erythropus, Bitis arietans, Dendroaspis viridis, Erythrocebus patas, Rattus rattus*, *Naja melanoleuca* and *Python sebae* . The other species are evaluated as less vulnerable.

## 4 Discussion

### 4.1 Diversity and the Use Importance Value of Wildlife Species

Habitat degradation and overexploitation remain the leading forces driving numerous species toward decline and local extinction (Maxwell et al. 2016). Unregulated wildlife exploitation and trade intensify these threats, posing significant challenges to global conservation efforts (Phelps et al. 2010; Coad et al. 2019; Cardoso et al. 2021; Roy and Kumar 2024). Wild animals, along with their body parts and derivatives, are hunted and traded for various purposes, including decoration, traditional medicine, food and the pet trade (D'Cruze, Assou, Coulthard, Norrey, et al. 2020; El Bizri et al. 2020; Harrington et al. 2021; Ingram et al. 2021). Wildlife use and trade remain among the most pressing threats to biodiversity conservation in West and Central Africa (e.g., Fa et al. 2002; Djagoun et al. 2018; Hema et al. 2019; Atsri et al. 2020; Ingram et al. 2021; Assou et al. 2021; Sonhaye‐Ouyé et al. 2022; Simo et al. 2023; Kaboumba et al. 2025). Our findings highlight a persistent gap between legislation and actual practices, a challenge widely documented across the region. Analyzing local perceptions provides valuable insights into identifying leverage points for effective conservation action, rather than serving as a justification for continued exploitation.

The use categories of wildlife species reported in the present study are consistent with previous studies on the usage of wildlife by local communities near protected areas in West and Central Africa (Ávila Martin et al. 2020; Ingram et al. 2021; Issifou et al. 2022; Sonhaye‐Ouyé et al. 2022; Simo et al. 2023). Both live wildlife and various parts are hunted and traded, providing protein and income for local populations and intermediaries involved in wildlife resources trade (Segniagbeto 2016; D'Cruze, Assou, Coulthard, Norrey, et al. 2020; D'Cruze, Harrington, Assou, Green, et al. 2020; Assou et al. 2021). The primary use of wildlife species in AWR is for food, which is essential for the nutritional balance of local populations. This is consistent with findings from the Abdoulaye Wildlife Biosphere Reserve in Togo (Issifou et al. 2022). Unlike that study, however, artisanal use is not significantly represented among the local populations bordering AWR.

The IVIUsp is a reliable parameter for characterizing the importance of biodiversity use (Atakpama et al. 2021). The importance of a species is determined not only by its frequency of citations or the number of people using it but also by the number of specific uses. Reptiles, alongside mammals, are among the most valued animal species by the neighboring populations of the AWR. Mammals remain the most popular species due to their diverse use categories and specific uses (Issifou et al. 2022). In addition to food, medicinal use is also highly reported and often includes reptiles. Mammals are the most frequently reported species probably due to their overall diversity in the AWR and the significant biomass they provide. According to Amegbenyuie et al. (2023), the abundance and availability of a resource are often correlated with knowledge of its uses.

### 4.2 Wildlife Species Vulnerability

Indices of local vulnerability to use pressures indicate that no species in the AWR are classified as highly vulnerable. However, 14 species are identified as moderately vulnerable to use pressures. This could be explained by the AWR's relatively intact state, which allows it to continue serving as a biodiversity refuge (Woegan et al. 2013; MERF 2017). Unlike other protected areas in Togo, such as the Fosse aux Lions‐Fosse de Doungh complex (Atakpama et al. 2023), the Oti‐Kéran‐Mandouri complex in ecological zone I (Polo‐Akpisso et al. 2018), and the Amou‐Mono Classified Forest in ecological zone V (Kokou et al. 2023), the AWR appears to be less significantly impacted by human activities. Among the 14 moderately vulnerable species, four are globally threatened according to the IUCN. This underscores the need for additional data collection on wildlife populations to better assess their vulnerability and implement more effective biodiversity management programs. Sustainable management and the promotion of biodiversity, particularly through tourism, can generate income (Koumantiga et al. 2021) and raise awareness about the importance of wildlife conservation.

## 5 Conclusion

This study identified the diversity, use‐importance value and vulnerability of the AWR's wildlife species. A total of 49 species, primarily used for food, were reported. Among these, ungulates, particularly Bovidae, are the most diverse common taxon. *Erythrocebus patas* , *Naja melanoleuca* , *Dendroaspis viridis*, *Python regius* and *Bitis arietans* have the highest use‐importance values. According to the local vulnerability index, 14 species are moderately vulnerable to use pressures within the AWR. Sustainable management of wildlife and its habitat is essential for conserving animal diversity. Promoting domestic animal breeding among local communities could increase the availability of animal protein and provide alternative sources of income, thereby reducing pressure on the AWR's wildlife resources. Additionally, strengthening law enforcement, engaging communities through alternative livelihoods, and raising awareness about the importance of conserving and restoring forest ecosystems and wildlife resources are essential.

## Author Contributions

**Wiyaou Borozi:** conceptualization (equal), data curation (lead), formal analysis (equal), funding acquisition (lead), investigation (equal), methodology (equal), resources (equal), software (equal), visualization (equal), writing – original draft (lead), writing – review and editing (lead). **Wouyo Atakpama:** conceptualization (equal), data curation (equal), formal analysis (equal), investigation (equal), methodology (lead), project administration (equal), software (equal), supervision (equal), validation (equal), writing – original draft (equal), writing – review and editing (lead). **Delagnon Assou:** validation (equal), writing – review and editing (equal). **Armand Kuyema Natta:** conceptualization (equal), investigation (equal), methodology (equal), project administration (lead), supervision (lead), validation (lead), writing – original draft (equal), writing – review and editing (equal).

## Funding

This work was supported by the International Tropical Timber Organization.

## Conflicts of Interest

The authors declare no conflicts of interest.

## Data Availability

I confirm that access to all necessary data files is provided to editors and reviewers.
